# An ecosystem-scale perspective of the net land methanol flux: synthesis of micrometeorological flux measurements

**DOI:** 10.5194/acp-15-7413-2015

**Published:** 2015-01-27

**Authors:** G. Wohlfahrt, C. Amelynck, C. Ammann, A. Arneth, I. Bamberger, A. H. Goldstein, L. Gu, A. Guenther, A. Hansel, B. Heinesch, T. Holst, L. Hörtnagl, T. Karl, Q. Laffineur, A. Neftel, K. McKinney, J. W. Munger, S. G. Pallardy, G. W. Schade, R. Seco, N. Schoon

**Affiliations:** 1 Institute of Ecology, University of Innsbruck, Innsbruck, Austria; 2 European Academy of Bolzano, Bolzano, Italy; 3 Belgian Institute for Space Aeronomy, Brussels, Belgium; 4 Research Station Agroscope, Climate and Air Pollution Group, Zürich, Switzerland; 5 Karlsruhe Institute of Technology, IMK-IFU, Garmisch-Partenkirchen, Germany; 6 Institute of Agricultural Sciences, ETH Zürich, Zürich, Switzerland; 7 Department of Environmental Science, Policy, and Management, University of California, Berkeley, CA, USA; 8 Environmental Sciences Division, Oak Ridge National Laboratory, Oak Ridge, TN, USA; 9 Atmospheric Sciences and Global Change Division, Pacific Northwest National Laboratory, Richland, WA, USA; 10 Institute of Ion Physics and Applied Physics, University of Innsbruck, Innsbruck, Austria; 11 Exchanges Ecosystems-Atmosphere, Department Biosystem Engineering (BIOSE), University of Liege, Gembloux, Belgium; 12 Department of Physical Geography and Ecosystem Science, Lund University, Lund, Sweden; 13 Institute of Meteorology and Geophysics, University of Innsbruck, Innsbruck, Austria; 14 Royal Meteorological Institute, Brussels, Belgium; 15 School of Engineering and Applied Sciences, Harvard University, Cambridge, MA, USA; 16 Department of Forestry, University of Missouri, Columbia, MO, USA; 17 Department of Atmospheric Sciences, Texas A&M University, College Station, TX, USA; 18 Department of Earth System Science, University of California, Irvine CA 92697, USA

## Abstract

Methanol is the second most abundant volatile organic compound in the troposphere and plays a significant role in atmospheric chemistry. While there is consensus about the dominant role of living plants as the major source and the reaction with OH as the major sink of methanol, global methanol budgets diverge considerably in terms of source/sink estimates reflecting uncertainties in the approaches used to model, and the empirical data used to separately constrain these terms. Here we compiled micrometeorological methanol flux data from eight different study sites and reviewed the corresponding literature in order to provide a first cross-site synthesis of the terrestrial ecosystem-scale methanol exchange and present an independent data-driven view of the land–atmosphere methanol exchange. Our study shows that the controls of plant growth on the production, and thus the methanol emission magnitude, and stomatal conductance on the hourly methanol emission variability, established at the leaf level, hold across sites at the ecosystem-level. Unequivocal evidence for bi-directional methanol exchange at the ecosystem scale is presented. Deposition, which at some sites even exceeds methanol emissions, represents an emerging feature of ecosystem-scale measurements and is likely related to environmental factors favouring the formation of surface wetness. Methanol may adsorb to or dissolve in this surface water and eventually be chemically or biologically removed from it. Management activities in agriculture and forestry are shown to increase local methanol emission by orders of magnitude; they are however neglected at present in global budgets. While contemporary net land methanol budgets are overall consistent with the grand mean of the micrometeorological methanol flux measurements, we caution that the present approach of simulating methanol emission and deposition separately is prone to opposing systematic errors and does not allow taking full advantage of the rich information content of micrometeorological flux measurements.

## 1 Introduction

Methanol (CH_3_OH) is, on average, the second most abundant volatile organic compound (VOC) in the troposphere (Jacob et al., 2005), with typical mole fractions in the continental boundary layer of 1–10 nmolmol^−1^ (Heikes et al., 2002). With an atmospheric lifetime of 5–12 days (Jacob et al., 2005), methanol has been shown to play a role in modulating the presence of oxidants in the upper troposphere (Tie et al., 2003). It affects atmospheric chemistry as an atmospheric source of formaldehyde (Palmer et al., 2003) and carbon monoxide (Duncan et al., 2007). Model calculations suggest methanol emissions constitute 10 % of the total global biogenic non-methane VOC (BVOC) emissions, the second highest single compound contribution after isoprene (Guenther et al., 2012).

The primary source of atmospheric methanol is emissions from living plants, followed by smaller source contributions from the decay of dead plant matter, biomass burning, direct emissions from anthropogenic activities, the ocean and atmospheric production (Seco et al., 2007). The major sink for methanol is oxidation by OH radicals, followed by dry and wet deposition to land and ocean. Estimates of the global land net flux, i.e. the balance between sources and sinks of methanol on land, vary widely between 75–245 Tgy^−1^ (Singh et al., 2000; Galbally and Kirstine, 2002; Heikes et al., 2002; Tie et al., 2003; von Kuhlmann et al., 2003a, b; Millet et al., 2008; Stavrakou et al., 2011), although more recent estimates converge to a more narrow range of 75–108 Tgy^−1^ (Jacob et al., 2005; Millet et al., 2008; Stavrakou et al., 2011).

Much of the knowledge and data embedded into the parameterisation of plant methanol emissions derives from work at the leaf level (Galbally and Kirstine, 2002; Guenther et al., 2012). In living plants, methanol is produced as a by-product of pectin metabolism during cell wall synthesis (Fall and Benson, 1996) and methanol production and emission thus are positively correlated with plant growth (Custer and Schade, 2007; Hüve et al., 2007) and pectin content (Galbally and Kirstine, 2002). This circumstance led Galbally and Kirstine (2002) to simulate global methanol emissions as a function of net primary productivity (NPP) that consists of pectin and the fraction thereof which is demethylated during growth, an approach which later has been adopted by others (Jacob et al., 2005; Millet et al., 2008). Most other global budgets rely on the MEGAN model (Guenther et al., 1995, 2012) to simulate methanol emissions using light and temperature-driven algorithms. While lacking a sound physiological basis, the latter approach is successful in simulating observed variations in methanol emissions due to the fact that methanol emissions are strongly controlled by stomatal conductance, reflecting its low Henry constant (Niinemets and Reichstein, 2003; Harley et al., 2007). Stomatal conductance, in the absence of soil water limitations, tracks diurnal variations in light and temperature, which in turn correlate with diurnal methanol emissions (e.g. Hörtnagl et al., 2011).

The deposition of methanol in global models is typically represented in a very simplistic fashion using fixed deposition velocities. These vary by up to a factor of four between different studies (e.g. Galbally and Kirstine, 2002; Millet et al., 2008) and are often, constrained by observed atmospheric concentrations, tuned to close the atmospheric budget. Recently, several studies have reported significant methanol deposition to terrestrial ecosystems and/or clear evidence of bidirectional exchange (Misztal et al., 2011; Schade et al., 2011; Laffineur et al., 2012). The observed deposition has been related to high ambient methanol mole fractions downwind of industrial methanol sources (Laffineur et al., 2012), the presence of water films in the plant canopy or soil within which methanol may adsorb/dissolve and can be removed by chemical transformations (Laffineur et al., 2012) and/or methylotrophic bacteria (Fall and Benson, 1996; Abanda-Nkpwatt et al., 2006).

In summary, while there is consensus about the dominant role of living plants as the major source and the reaction with OH radicals as the major sink of methanol, global methanol budgets diverge considerably in terms of source/sink estimates (Jacob et al., 2005) reflecting uncertainties in the approaches used in models and the empirical data used to separately constrain the source/sink terms.

Micrometeorological methods allow measurements of the net exchange of mass, energy and momentum between the underlying surface and the atmosphere over the spatial scale of typically hundreds of meters (Baldocchi et al., 1988). Thanks to advances in proton-transfer-reaction mass spectrometry, a fast and sensitive analytical method to determine methanol mole fractions in ambient air in real-time during the past decade (Karl et al., 2001, 2002; Müller et al., 2010), ecosystem-scale methanol flux measurements have been reported from multiple sites and in a few cases over multiple seasons (Tables 1 and 2). Because micrometeorological flux measurements allow quantification of the net flux of methanol between ecosystems and the atmosphere quasi-continuously and over extended periods of time, they are ideal for assessing the performance of models at the ecosystem scale. Up to now, however, few (if any) studies have made use of this rich data source in a more holistic fashion.

The main objective of this study is thus (i) to compile the available ecosystem-scale methanol exchange data from micrometeorological flux measurements, (ii) to conduct a first cross-site synthesis of the magnitude of and controls on the terrestrial net ecosystem methanol exchange and (iii) to provide an independent constraint on the land methanol exchange against which models can be compared.

## 2 Methods

In total, growing season data from eight sites in the Northern Hemisphere were available for the present synthesis (Table 1). Key metrics of micrometeorological methanol flux measurements from additional sites were obtained from a literature survey (Table 2). The climate space covered the Mediterranean to the Boreal climate zone, with mean annual temperatures ranging from −0.7 to +9.0 °C, however most of the sites (six) were located in the Temperate climate zone. The study sites comprised four forests, three managed grasslands and one wetland.

The net ecosystem methanol exchange was determined by means of the so-called virtual disjunct eddy covariance (vDEC) method (Karl et al., 2002) at seven sites and by the relaxed eddy accumulation (REA) method at one site. With the vDEC method, as with the “true” eddy covariance method (Baldocchi et al., 1988), measurements of the three-dimensional wind vector by means of sonic anemometers are made at high temporal resolution (50–100 ms). Methanol mole fractions are measured at disjunct time intervals separated typically by 1–3 s with integration times of 100–500 ms (Table S1). As shown by Hörtnagl et al. (2010), the vDEC method increases random variability compared to the true eddy covariance method, but does not result in a systematic bias. This was confirmed by a direct comparison between vDEC and true eddy covariance methanol flux measurements by Müller et al. (2010). Methanol mole fractions were measured with proton-transfer-reaction mass spectrometers (PTR-MS) on mass-to-charge ratio (*m/z*) 33 (see Hansel et al. (1995), Lindinger et al. (1998) and Graus et al. (2010) for more details on the PTR-Q-MS and PTR-TOF-MS technology). The PTR-MS instruments were typically housed in a sheltered location some distance away or at the bottom of the instrument tower supporting the sonic anemometer. Air was pumped from an inlet close to the sonic anemometer to the PTR-MS through an inlet line, which was designed to minimise interactions between the tubing material and methanol (i.e. through use of inert materials and heating). Further details on the study sites, instrumentation and experimental protocols are given in Tables 1 and S1 and the references cited therein. In contrast to the eddy covariance CO_2_ flux community (Baldocchi, 2003), which has made considerable progress in standardising flux measurement protocols (Mauder and Foken, 2006), little effort has been made in the (much smaller) VOC flux community to standardise measurement protocols. In the present study we have decided to use the data from the different sites as they are, with measurements, processing and quality controlled as described in the key references in Table 1. We acknowledge that this approach potentially introduces systematic bias among sites. As shown in Table S1 in the Supplement, there are necessarily large differences in the air sampling systems due to different canopy and tower heights, but the PTR-MS setups were remarkably similar.

At the Blodgett Forest study site, methanol exchange was determined with the relaxed eddy accumulation (REA) method by sampling up- and down-drafts of air into separate reservoirs (cooled activated carbon microtraps), which were analysed immediately after collection by a gas chromatography flame ionisation detector technique (Schade and Goldstein, 2001). Even though the REA method is a less direct method than the vDEC (Hewitt et al., 2011), the data from Blodgett Forest were included in the present analysis because several studies demonstrated good correspondence between VOC fluxes measured concurrently by the REA and the eddy covariance method (e.g. Westberg et al., 2001; Lee et al., 2005).

Additional auxiliary data included concurrent measurements of the major environmental drivers, including air temperature and humidity, horizontal wind speed, incident photosynthetically active radiation and precipitation above the canopy and soil temperature and water content in the near-surface soil. In addition we collected above-canopy net ecosystem carbon dioxide exchange (NEE), which was measured at each site within the frame of the FLUXNET project (Baldocchi et al., 2001; Baldocchi, 2003), and derived therefrom gross photosynthesis (GPP) and ecosystem respiration (Reichstein et al., 2005).

Data were brought to a common format and analysed with SPSS version 19. Statistical analysis was performed, if not stated otherwise, on the quality filtered half-hourly data.

## 3 Results and discussion

### 3.1 Magnitude of methanol exchange

**Figure 1:**
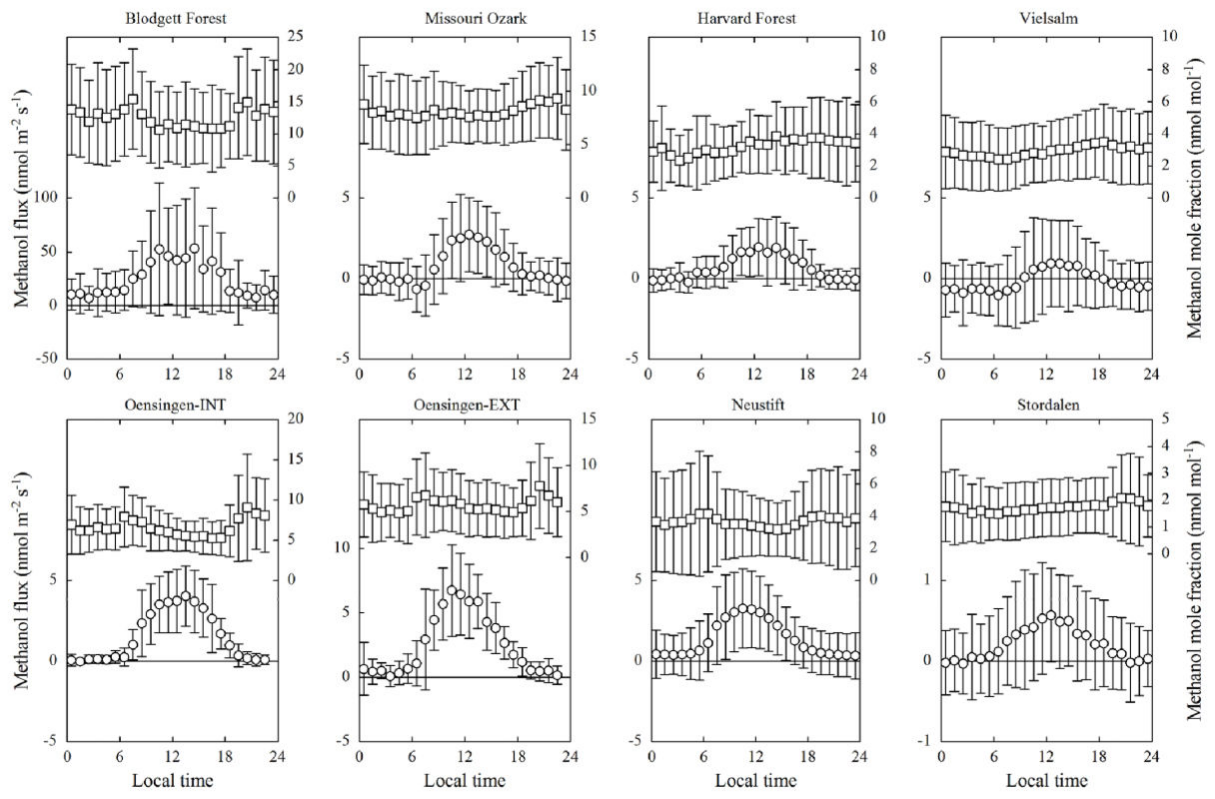
Hourly bin-averaged diurnal variation of methanol fluxes (circles; left *y* axis) and mole fractions (squares; right *y* axis) at the eight study sites (error bars represent ± one SD). Note the differing scaling on the *y* axis. Data from Oensingen-INT, Oensingen-EXT and Neustift are exclusive of periods influenced by management practises.

**Figure 2:**
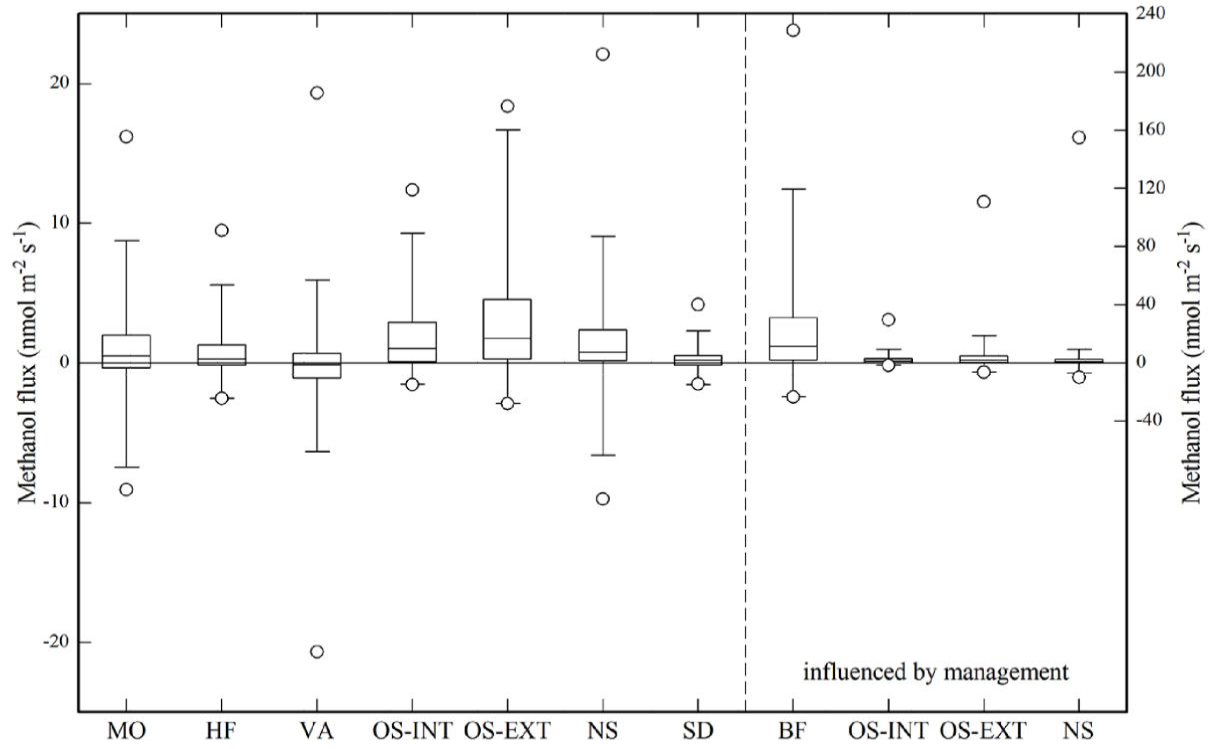
Box-plots of methanol fluxes at the eight study sites. The left *y* axis refers to sites/measurements not influenced by site management events, while the right *y* axis (note differing scaling) shows data for Blodgett Forest and the grassland sites inclusive of measurements during/after management (MO – Missouri Ozark, HF – Harvard Forest, VA – Vielsalm, OS-INT – Oensingen-Intensive, OS-EXT – Oensingen-Extensive, NS – Neustift, SD – Stordalen, BF – Blodgett Forest). Box plots show minima/maxima (circles), 5 and 95 % quartiles (whiskers), the interquartile range (box) and the median (horizontal line).

**Figure 3:**
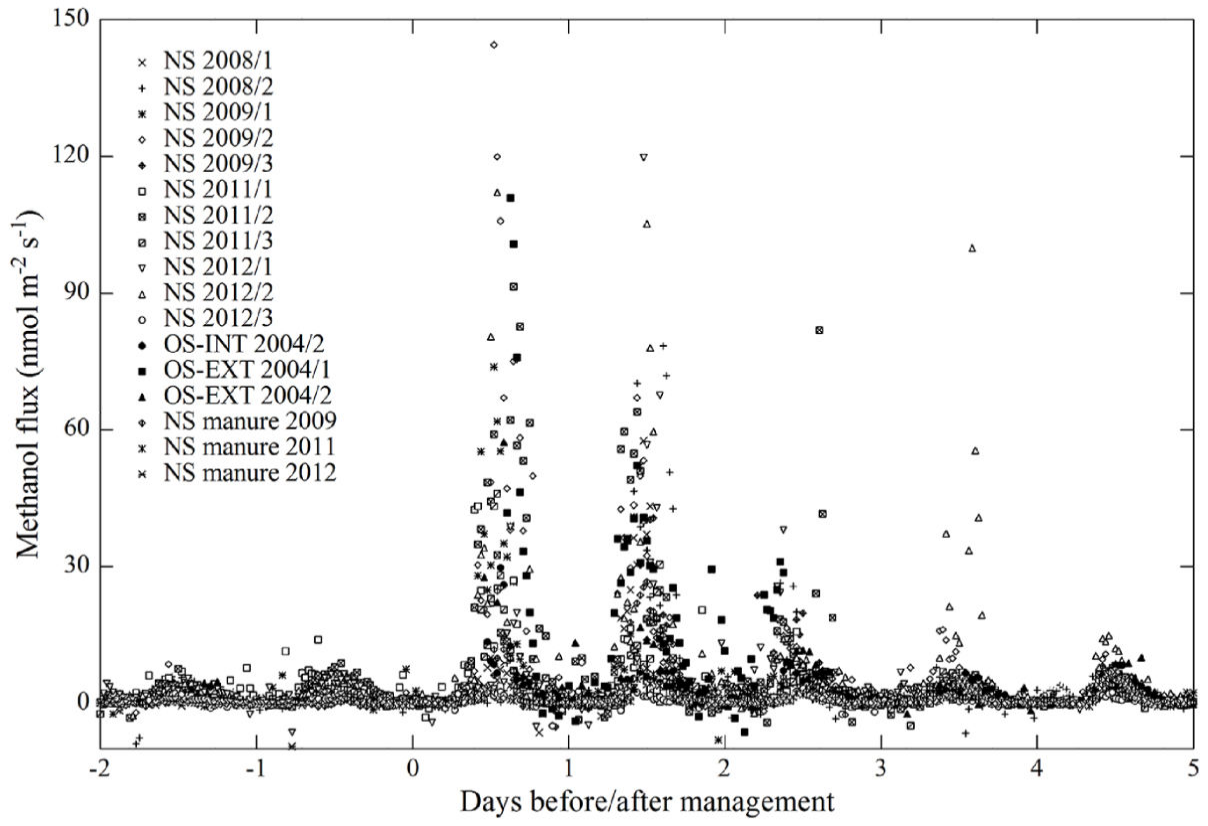
Effect of management (harvest and manure application) on methanol fluxes of grassland study sites Neustift (NS), Oensingen-INT (OS-INT) and Oensingen-EXT (OS-EXT) with indication of study year and, where applicable, number of harvest.

The eight investigated study sites, as shown in Figs. 1 and 2 and Table 2, showed quite contrasting methanol exchange rates, however, also exhibited common features: all study sites showed both net emission and net deposition of methanol (Fig. 2) and methanol fluxes exhibited a more or less pronounced average diurnal pattern (Fig. 1), in phase with the diurnal course of incident radiation and air temperature (Fig. S1). Flux magnitudes were however quite different: by far the largest net emissions were observed at Blodgett Forest, whose average methanol emissions (23.9 nmolm^−2^ s^−1^) exceeded those of the other sites by a factor of 10 and more (Table 2). The three grasslands, excluding periods following management activities, were characterised by average net emission rates of 1.5–2.8 nmolm^−2^ s^−1^. Management, harvesting and the application of organic fertiliser, caused methanol emissions from the grasslands to increase by an order of magnitude during the day of the management intervention and remain elevated a few days thereafter, before fluxes returned back to previous values (Fig. 3). These were followed by the Missouri Ozark and Harvard Forest mixed forest sites (0.7–0.9 nmolm^−2^ s^−1^). The lowest average methanol fluxes were measured at the wetland site of Stordalen (0.2 nmolm^−2^ s^−1^) and the mixed forest of Vielsalm. The latter in fact was characterised by a negative average flux (−0.1 nmolm^−2^ s^−1^), i.e. methanol deposition exceeded emissions at this site.

From a comparison with the other seven study sites (Fig. 2) and the literature (Table 2) it becomes clear that the emissions observed at Blodgett Forest are exceptionally high, even compared to elevated emissions observed over agricultural crops and grasslands after harvesting or the application of organic fertiliser (e.g. Brunner et al., 2007; Davison et al., 2008; Hörtnagl et al., 2011; Ruuskanen et al., 2011; Brilli et al., 2012). Schade and Goldstein (2001) attributed these high emissions to the cutting of shrubs in the understory, such as manzanita, of the site prior to the measurements, as part of a regular forest plantation management intervention. The cut plant material was left at the site and may have caused the elevated methanol emissions, similar to what was observed at the grassland sites after harvesting (Fig. 3). In contrast to the grassland sites, where these emissions were confined to less than three days after harvesting (Fig. 3) and cuttings were removed later, elevated emissions at Blodgett Forest were sustained. Bouvier-Brown et al. (2012) noted that measurements in subsequent years showed lower fluxes by a factor of 2–3. Park et al. (2014) measuring BVOC fluxes at Blodgett Forest ten years later with the vDEC method reported an average methanol flux of 4.2 nmolm^−2^ s^−1^, which is comparable in magnitude with the results from the other sites of this study and non-urban sites in the literature (Table 2). Park et al. (2014) also measured vDEC 2-Methyl-3-butene-2-ol (MBO) fluxes, which agreed with the corresponding REA flux estimates measured in 1999 concurrently with the methanol fluxes by Schade and Goldstein (2001). We are thus confident that the observed large emissions at Blodgett forest likely reflected the recent disturbance of the site.

Large net deposition fluxes of methanol, and even sites that represent net methanol sinks over extended periods of time, have not been reported in the literature until very recently (Langford et al., 2010a; Misztal et al., 2011; Schade et al., 2011; Laffineur et al., 2012). The present study confirms that net deposition of methanol is a common phenomenon (Table 2), which is observed at half of the study sites for more than 25 % of the time (Fig. 2). Laffineur et al. (2012) developed a theoretical framework to simulate methanol exchange at Vielsalm and showed that the bi-directional nature of methanol exchange can be explained by adsorption/desorption of methanol in water films within the ecosystem (aided by the low Henry constant of methanol) and a postulated sink process. While the latter had to be invoked in order to make the model match the sustained deposition fluxes, it is well established that methylotrophic bacteria inhabit plant surfaces and soils (Conrad, 1996; Fall and Benson, 1996; Conrad and Claus, 2005; Kolb, 2009; Stacheter et al., 2013) and may significantly reduce net leaf and ecosystem methanol emissions (Abanda-Nkpwatt et al., 2006).

After excluding data from Blodgett Forest and the grassland data influenced by management activities, we calculate a “grand mean” of 1 nmolm^−2^ s^−1^ as the average of the methanol fluxes of all sites in this study. Assuming the Earth’s ice-free land area (133.8×10^12^ m^2^) to emit methanol at this average rate year-round, which is an overestimation due to off-season fluxes being typically much lower than the growing season data compiled in this study (Bamberger et al., 2014), extrapolates to a global net land methanol flux of 135 Tgy^−1^. This value falls into the middle of the range of available global budget studies (75–245 Tgy^−1^; Table 2) and is quite close to the 75–108 Tgy^−1^ range of budgets published after 2005 (Jacob et al., 2005; Millet et al., 2008; Stavrakou et al., 2011). In addition to a likely warm-season bias, globally important ecosystems, such as tropical forests, are under-represented in our study, and included sites are likely not representative of pectin contents elsewhere (Custer and Schade, 2007). We thus stress the large uncertainties associated with this simplistic up-scaling.

**Figure 4:**
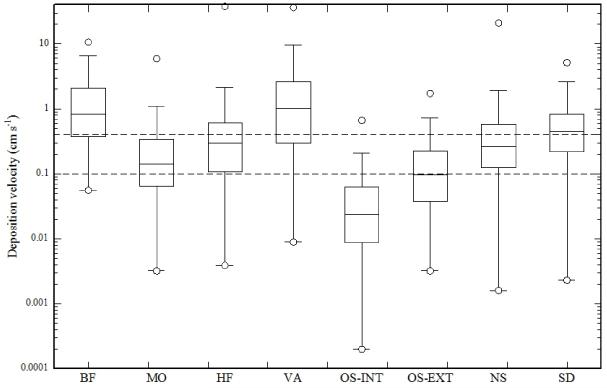
Box-plots of nighttime methanol deposition velocities at the eight study sites. Horizontal dashed lines indicate the range of deposition velocities (0.1–0.4 cm s^−1^) used in global budgets (see also Table 2). Box plots show minima/maxima (circles), 5 and 95 % quartiles (whiskers), the interquartile range (box) and the median (horizontal line).

Observed nighttime net deposition velocities (medians) ranged between 0.02 and 1.0 cms^−1^, with five of the eight sites bracketing the range of 0.1–0.45 cms^−1^ (Fig. 4). Including daytime deposition flux measurements did not substantially change these ranges (compare Fig. 4 with Fig. S2). These values are consistent with nighttime deposition velocities reported in the literature (Table 2) and overlap with the range of fixed deposition velocities of 0.1–0.4 cms^−1^ used in global methanol budgets (Singh et al., 2000; Galbally and Kirstine, 2002; Heikes et al., 2002; von Kuhlmann et al., 2003a, b; Jacob et al., 2005; Millet et al., 2008). Due to the concurrent emission and deposition of methanol these observed deposition velocities represent “net” deposition velocities, while values used in global budget studies are “gross” deposition velocities. Because the former are lower than the latter if there is any concurrent emission of methanol, this suggests that global models may be underestimating land deposition velocities and thus, provided that models correctly reproduce atmospheric concentrations, may be underestimating methanol sources to a similar degree.

Methanol mole fractions at the height of the flux measurements (Table 1) exhibited relatively little diurnal variability, with a tendency towards minima during daylight periods and the afternoon (Fig. 1). The highest (median) mole fractions were found at Blodgett Forest (11.6 nmolmol^−1^), the lowest at Stordalen (1.4 nmolmol^−1^), consistent with the range of 1–10 nmolmol^−1^ reported by Heikes et al. (2002) for the continental boundary layer. Overall, mole fractions correlated positively with methanol fluxes across sites (*r*^2^ = 0.69, *p* = 0.011), i.e. higher ambient mole fractions were associated with larger net emissions.

### 3.2 Controls on methanol exchange

In order to investigate the controls on methanol exchange, a multiple linear regression analysis was conducted for each site, separating the flux data by their sign, i.e. into net deposition and net emission (Table 3).

Methanol emission scaled positively with incident photosynthetically active radiation and evapotranspiration and these two independent variables explained the highest fraction of the variance (0.17 < *r*^2^ < 0.62; *p* < 0.001) at most sites. We interpret this to indicate the strong stomatal control of methanol exchange, owing to the low Henry constant which favours leaf-internal partitioning of methanol to the liquid phase (Niinemets and Reichstein, 2003), rather than a light-effect, since Oikawa et al. (2011b) have shown that methanol emissions are not directly affected by light.

**Figure 5:**
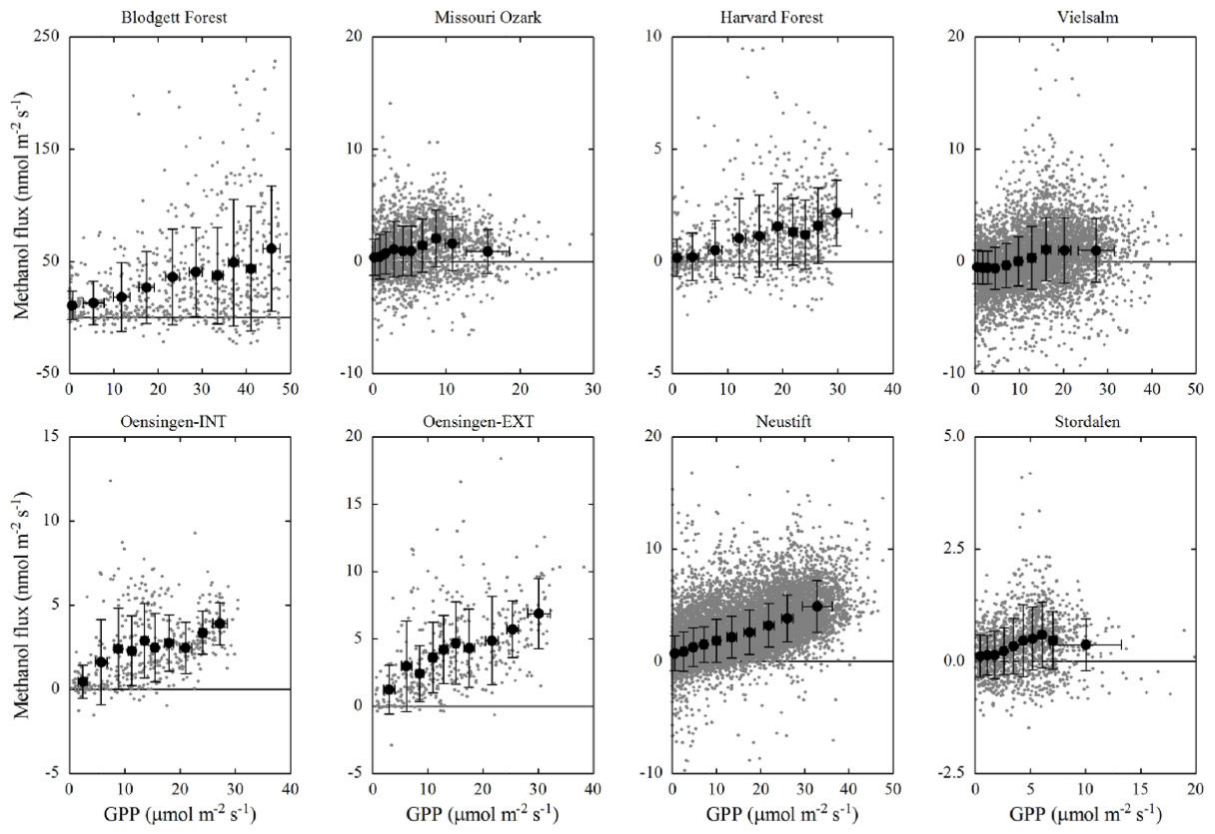
Relationship between gross photosynthesis (GPP) and methanol flux. Small grey symbols represent half-hourly flux measurements, black closed symbols 10 bin averages with equal numbers of data. Error bars refer to one SD. Note different *x* and *y* scales in different panels.

GPP and air temperature, which explained 7 to 43 % (*p* < 0.001) of the variability at the individual sites (Table 3), were positively related to methanol emissions, which we interpret to indicate a general relationship of these two variables with plant growth and thus methanol production. GPP provides assimilates for growth and temperature tightly controls cell division and enzyme reaction rates. While this results in correlations between methanol emission and these factors, actual methanol production has been shown to be more complex (Harley et al., 2007; Oikawa et al., 2011a) and these relationships should thus be viewed as phenomenological. Galbally and Kirstine (2002) were the first to link plant growth and methanol emissions in a global budget by assuming proportionality with NPP. Here we use GPP, which equals NPP plus autotrophic respiration, as an alternative proxy for plant growth that was generally available in the present data set, and the corresponding relationships with net methanol fluxes are shown in Fig. 5 (Fig. S3 in the Supplement shows the relationships with the net ecosystem CO_2_ exchange). Slopes of linear regressions (forced through the origin; excluding Blodgett Forest and grassland data affected by management activities) ranged between 3.5 × 10^−5^ (Vielsalm) and 2.5 × 10^−4^ (Oesingen-EXT) gC-CH_3_OH gC-GPP^−1^, with an average of 1.25 × 10^−4^ gC-CH_3_OH gC-GPP^−1^.

**Figure 6:**
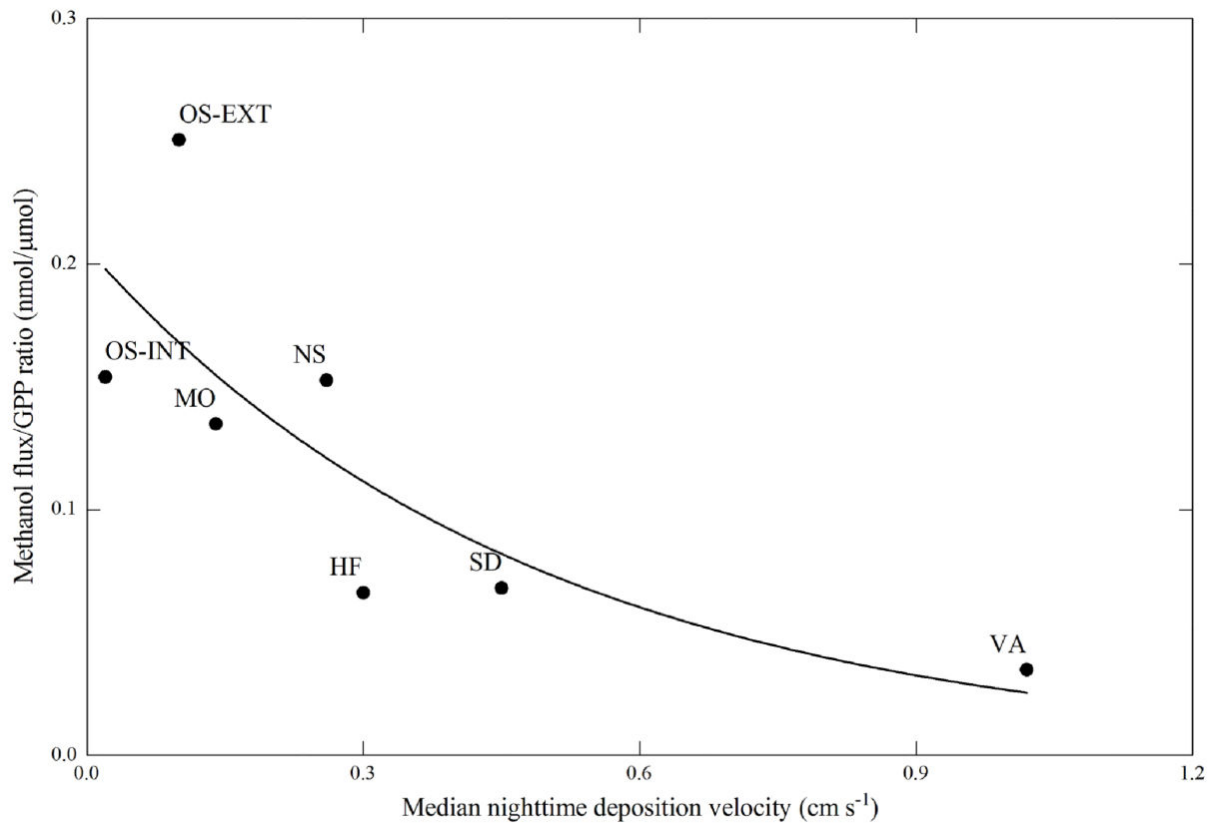
Methanol flux to GPP ratio as a function of the median nighttime deposition velocity. The solid line represents an exponential fit (*r*^2^ = 0.77).

Taking the most recent global GPP value (123 PgCy^−1^) from Beer et al. (2010) this yields a net land methanol flux of 41 Tgy^−1^, which is about half of the lowest estimates available from global budgets (Millet et al., 2008; Stavrakou et al., 2011). Accounting for the positive y-o set (i.e. not forcing the regression through the origin) observed at most sites (Fig. 5) or filtering data for positive methanol fluxes increases the above number by only 20 % (data not shown). Making the assumption that NPP amounts to around 50 % of GPP (Waring et al., 1998; Zhang et al., 2009) approximately doubles the average number quoted above. Compared to the range of 3.5–5.3 × 10^−4^ gC-CH_3_OH gC-NPP^−1^ deduced from the literature (Galbally and Kirstine, 2002; Millet et al., 2008; Stavrakou et al., 2011), our values of NPP lost as net land methanol flux are thus lower by about a factor of two. As shown in Fig. 6, an inverse relationship between the fraction of GPP that was lost as net methanol emission and the median nighttime deposition velocities was observed, with an exponential fit explaining 77 % of the variability between sites (excluding data from Blodgett Forest). In contrast, no significant correlation between the net methanol flux to GPP ratio was found with GPP itself (data not shown), suggesting no relationship between site productivity and the fraction of GPP that is lost as net methanol emission. The magnitude of methanol deposition thus clearly influences the observed fraction of GPP that is lost as methanol emission and limits the usefulness of GPP for up-scaling the net methanol exchange. In addition, it should be stressed that on short time scales GPP may be poorly correlated with NPP and even less with growth and the associated demethylation of pectin (Galbally and Kirstine, 2002).

Friction velocity and relative humidity explained slightly lower fractions of the variance compared to air temperature and GPP (Table 3). The positive relationship between friction velocity and methanol emission likely reflects the high degree of co-variation between friction velocity and air temperature and photosynthetically active radiation (data not shown). Relative humidity was inversely related to methanol emission at all sites (Table 3), which may result from canopy water films developing during periods of high relative humidity (Burkhardt et al., 2009) within which methanol may adsorb/dissolve, effectively resulting in a reduction of the net emission. Alternatively, this may reflect the inverse relationship of relative humidity with temperature and photosynthetically active radiation and their relationship with methanol exchange discussed above. The time since the end of the last precipitation event (TSEOP), which was introduced as a surrogate for the presence of canopy water films (Laffineur et al., 2012), and soil water content explained less than 8 % of the variability in methanol emissions (Table 3). In the case of TSEOP, this likely indicates that a more process-based approach would be required to properly capture the effect of wetting and subsequent drying on methanol exchange (Warneke et al., 1999; Laffineur et al., 2012).

The investigated independent variables generally explained a smaller fraction of the variability in observed deposition compared to emission fluxes and half of the relationships were statistically not significant (Table 3). Relative humidity and friction velocity were the independent variables explaining the highest fraction (up to 21 %) of the variance at most sites. Except for one site, friction velocity was negatively correlated with methanol deposition, suggesting more efficient downward transport of methanol as mechanical turbulence increases. In contrast to methanol emissions, which were inversely related to relative humidity, a positive correlation with methanol deposition was found at half of the sites, indicating that relative humidity plays a more variable role among sites in modulating deposition than emission. The remaining variables explained less than 10 % of the variability in observed methanol deposition fluxes (except for the intensive grassland of Oensingen).

In an attempt to investigate the common and site-specific controls on methanol emission and deposition, all data (except for Blodgett forest and those from the grassland sites influenced by management activities) were subjected to a univariate analysis of variance (Table 4). For methanol emissions, site identity and photosynthetically active radiation were the most important main effects. The largest fraction of variance was, however, explained by the interaction terms of site with relative humidity (*η*^2^ = 1.45 %) and GPP (*η*^2^ = 0.98 %), and to a lesser degree with photosynthetically active radiation and air temperature (Table 4). For methanol deposition, site identity was the only significant main factor (*η*^2^ = 2.96 %) and also contributed the largest fraction of explained variance, followed by the interaction terms between site and relative humidity and air temperature (Table 4). Overall this suggests that controls on methanol exchange are strongly site-specific and/or that factors not accounted for, such as soil type and microbial activity, play a substantial, possibly interactive, role in governing the ecosystem-atmosphere methanol exchange.

## 4 Conclusions

By compiling micrometeorological methanol flux data from eight different sites and by reviewing the corresponding literature, this study provides a first cross-site synthesis of the terrestrial ecosystem-scale methanol exchange and presents an independent, data-driven view of the land–atmosphere methanol exchange. Below we summarise the major findings, draw conclusions and make recommendations for future work: it is now unequivocal that at the ecosystem scale methanol exchange is bi-directional (Figs. 1 and 2, Table 2) and at some sites, deposition can even prevail over emission during extended periods of time (Langford et al., 2010a; Misztal et al., 2011; Laffineur et al., 2012). This finding is not new from the perspective of global methanol budgets, which do account for deposition to land and the oceans in addition to the OH sink, but emission and deposition are treated separately which likely results in inconsistencies (Singh et al., 2000; Galbally and Kirstine, 2002; Heikes et al., 2002; Tie et al., 2003; von Kuhlmann et al., 2003a, b; Jacob et al., 2005; Millet et al., 2008; Stavrakou et al., 2011). The prominent role of deposition is an emerging feature of ecosystem-scale measurements and is in contrast to leaf-level work that almost exclusively reported methanol emissions and focussed on describing the corresponding controls (e.g. Niinemets and Reichstein, 2003; Harley et al., 2007; Hüve et al., 2007).

The bi-directional nature of the terrestrial methanol flux makes it difficult for the present generation of models, which simulate emission and deposition separately, to fully capitalise on the rich information of micrometeorological measurements for calibration/validation. Guenther et al. (2012) proposed adding an estimate of the deposition flux to the net flux measured by micrometeorological methods to be used for calibrating the primary emission in MEGAN. While correct in principle, the emerging picture of methanol deposition being more difficult to predict than emission (Tables 3 and 4), makes it difficult in practice to “estimate” the magnitude of the deposition flux with confidence. We argue that these difficulties should be addressed by a new generation of models, which reflect the available process knowledge about the controls on both emission and deposition of methanol and merge it into a unified modelling framework. For the strong stomatal control on methanol emissions (Niinemets and Reichstein, 2003; Harley et al., 2007) and the role of water in adsorption/desorption of methanol (Laffineur et al., 2012), the corresponding theory is available. Land surface models which include a description of the ecosystem water budget, i.e. stomatal conductance, leaf energy balance, interception of precipitation (e.g. Berry et al., 1997), would provide most of the interfaces to this end. Further work is required in order to better understand the controls on leaf methanol production (Harley et al., 2007; Oikawa et al., 2011a), the role of chemical and/or biological removal of methanol on (wet) surfaces (Fall and Benson, 1996; Abanda-Nkpwatt et al., 2006; Laffineur et al., 2012) and the importance of soils as sources/sinks of methanol (Asensio et al., 2008; Greenberg et al., 2012; Stacheter et al., 2013; Peñuelas et al., 2014).

This (Fig. 3) and earlier work (Karl et al., 2001; Brunner et al., 2007; Davison et al., 2008; Hörtnagl et al., 2011; Ruuskanen et al., 2011; Brilli et al., 2012) conclusively show that management of agricultural ecosystems (biomass harvesting, grazing or application of organic fertiliser) results in short-term increases of methanol emissions by an order of magnitude. Despite being relatively short-lived, these bursts of BVOC emissions make a substantial contribution to the total BVOC budget of these agricultural ecosystems (Hörtnagl et al., 2011; Bamberger et al., 2014). Much less information is available for the effects of various forest management activities (pruning, thinning, clear-cut, residue management, etc.) on BVOC and methanol fluxes. Data from Blodgett Forest (Figs. 1 and 2) and the studies by Haapanala et al. (2012) and Schade and Goldstein (2003) suggest that forest management activities may cause longer-term perturbations of BVOC emissions compared to agricultural ecosystems. Given that the human appropriation of NPP has increased from 13 % of the NPP of potential vegetation in 1910 to 25 % in 2005 (Krausmann et al., 2013), we suggest that the effects of management on methanol emissions should be quantified for a larger range of ecosystems (in particular for managed forests) and be included in global budgets. As shown by Brilli et al. (2012) for grasslands, the magnitude of post-harvesting BVOC emissions scales with the amount of harvested biomass, suggesting that these emissions could be modelled based on agricultural/forestry census data (Schade and Goldstein, 2003), possibly in combination with remote sensing (for hindcast applications).

This study relied on data from eight study sites, reviewed additional 21 published studies and thus represents only a first step towards a data-driven assessment of the global land methanol flux. Data from additional sites in underrepresented ecosystem types and climates are required to better constrain differences between different ecosystem types which are embedded in model parameters of different plant functional types (PFT); e.g. at present ten of the eleven woody PFTs in MEGAN have one common methanol emission factor and the remaining five PFTs another one (Guenther et al., 2012). In a next step, methanol flux measurements need to be conducted over multiple years (including off-season periods; Bamberger et al., 2014) in order to be able to quantify and explain inter-annual variability in atmospheric methanol mole fractions. Building upon the experiences gathered in the FLUXNET project (Baldocchi et al., 2001), the BVOC flux community also should make a concerted effort towards standardising flux data acquisition and processing so that data are more readily comparable and models can be calibrated and validated based on harmonised data sets.

## Supplementary Material

SM

## Figures and Tables

**Table 1:** General characterisation of the study sites (see Table S1 for further details on experimental setup).

	Blodgett Forest (BF)	Missouri Ozark (MO)	Harvard Forest (HF)	Vielsalm (VA)	Oensingen INT (OS-INT)	Oensingen EXT (OS-EXT)	Neustift (NS)	Stordalen Mire (SD)
Country	USA	USA	USA	Belgium	Switzerland	Switzerland	Austria	Sweden
Latitude	38.89° N	38.76° N	42.54° N	50.30° N	47.28° N	47.28° N	47.12° N	68.33° N
Longitude	120.63° W	92.16° W	72.17° W	5.98° E	7.73° E	7.73° E	11.32° E	19.05° E
Elevation (m)	1315	216	340	450	450	450	970	351
MAP (mm)	1290	1110	1066	1000	1100	1100	852	304
MAT (°C)	9.0	13.6	7.8	7.5	9.0	9.0	6.5	−0.7
Climate	Mediterranean	Temperate continental	Temperate	Temperate maritime	Temperate continental	Temperate continental	Temperate alpine	Boreal
Plant functional type	Coniferous evergreen forest	Deciduous broadleaf forest	Mixed forest	Mixed forest	Grassland	Grassland	Grassland	Wetland
Management	Understory cut	–	–	–	Harvest	Harvest	Harvest	–
LAI(m^2^m^−2^)	1–1.7	1.3–3.5	4.8–5.4	2.6–3.8	0.4–3.5	0.2–5.1	0.2–7.8	up to 3.5
Measurement/avg. canopy height (m)	11/5	29/20	30/23	52/30	1.2/0.15	1.2/0.2	2.5/< 1.0	2.95/< 0.5
Data coverage from-to DOY (year)	142–170 (1999)	125–296 (2012)	149–248 (2007)	182–304 (2009) 60–273 (2010) 91–334 (2011)	176–213 (2004)	158–175 (2004) 214–249 (2004)	143–325 (2008) 78–305 (2009) 77–346 (2011) 87–330 (2012)	121–273 (2006) 121–260 (2007)
Flux method	REA	vDEC	vDEC	vDEC	vDEC	vDEC	vDEC	vDEC
Key reference	Schade and Goldstein (2001)	Seco et al. (unpublished)	McKinney et al. (2011)	Laffineur et al. (2012)	Brunner et al. (2007)	Brunner et al. (2007)	Hörtnagl et al. (2011)	Holst et al. (2010)

Abbreviations: MAP – mean annual precipitation, MAT – mean annual temperature, LAI – leaf area index.

**Table 2:** Literature survey of micrometeorological methanol flux studies and the net land methanol flux derived from global budget studies compared to the results of the present study.

			Methanol flux (nmolm^−2^s^−1^)					*V*_d_^a^ (cms^−1^)
	Vegetation type	Method	Average	SD	Median	Maximum	Minimum	
**Ecosystem-scale studies**								
Schade and Custer (2004)	bare agricultural soil	EC				4.6	0.0	0.1–0.4
Custer and Schade (2007)	Rye grass	EC	0.22	0.22	0.1	1.5	−0.6	~ 0.1
Warneke et al. (2002)	Alfalfa crop	DEC	4.7			34.7	0.0	
Schade et al. (2011)	Deciduous forest	REA				5.0	−3.6	1.1
Karl et al. (2003)	Mixed deciduous forest	vDEC	6.1			19.9	−1.7	
Spirig et al. (2005)	Mixed deciduous forest	vDEC				4.0	−1.1	
Baker et al. (2001)	Coniferous forest	REA				56.0	−12.0	
Karl et al. (2005)	Coniferous forest	vDEC	2.8	0.9				1.0
Rinne et al. (2007)	Coniferous forest	vDEC	1.4			3.7	0.1	
Park et al. (2014)	Pine forest	vDEC	4.2					
Karl et al. (2004)	Tropical rainforest	vDEC				4.8	−0.9	0.3
Langford et al. (2010a)	Tropical rainforest	vDEC	−0.3	2.6	−0.6			
Davison et al. (2009)	Mediterranean macchia	vDEC			3.7			
Park et al. (2013)	Orange orchard	EC	1.7					
Fares et al. (2012)	Citrus orchard	vDEC			0.26–2.74	10.0	−5.0	
Brilli et al. (2014)	SRC poplar plantation	EC	1.4		1.0			
Misztal et al. (2011)	Oilpalm plantation	vDEC	−0.4	0.9	−0.2	3.0	−3.1	
Velasco et al. (2005)	Urban	vDEC	9.0					
Langford et al. (2009)	Urban	(v)DEC	4.7	6.2	4.3			
Velasco et al. (2009)	Urban	vDEC	12.8	6.3				
Langford et al. (2010b)	Urban	vDEC	8.3	8.1	8.2			

**Global average net land flux** ^b^								
Heikes et al. (2002)			1.8					0.4
Galbally and Kirstine (2002)			0.7					0.1
Tie et al. (2003)			1.3					
Jacob et al. (2005)			0.8					0.2
Millet et al. (2008)			0.6					0.4
Stavrakou et al. (2011)			0.6					

**This study**								
Blodgett Forest	Coniferous forest	REA	23.9	36.9	11.3	228.7	−23.1	1.8
Missouri Ozark	Deciduous forest	vDEC	0.9	2.1	0.5	16.2	−9.0	0.3
Harvard Forest	Mixed deciduous forest	vDEC	0.7	1.5	0.3	9.5	−2.5	1.0
Vielsalm	Mixed deciduous forest	vDEC	−0.1	2.2	−0.1	19.3	−20.7	1.9
Oensingen-INT^c^	Grassland	vDEC	1.7(1.9)	2.0(2.6)	1.0(1.1)	12.4(29.8)	−1.5(−1.5)	0.1
Oensingen-EXT^c^	Grassland	vDEC	2.8(4.4)	3.1(9.0)	1.7(2.0)	18.4(110.9)	−2.9(−6.3)	0.2
Neustift^c^	Grassland	vDEC	1.5(1.8)	2.1(4.2)	0.8(0.8)	22.1(155.1)	−9.7(−9.7)	0.5
Stordalen	Wetland	vDEC	0.2	0.6	0.2	4.2	−1.5	0.7

a average nighttime deposition velocity

b the net land flux was derived by summing emissions from plants, decay of plant matter, biomass burning, anthropogenic activities and subtracting dry and wet deposition to land, dividing by the land area (133.8 × 10^12^ m^2^) and converting from mass to molar basis using 32gmol^−1^

c values in parenthesis include data influenced by site management events.

**Table 3:** Pearson correlation coefficients of multiple linear regressions of half-hourly methanol emission and deposition fluxes as a function of several independent variables (PAR – photo-synthetic photon flux density, RH – relative air humidity TA – air temperature, SWC soil water content, u_*_, – friction velocity ET – evapotranspiration, GPP – gross primary productivity, TSEOP – time since end of precipitation, *n* – number of measurements).

	Emission							
	BF	MO	HF	VA	OS-INT^a^	OS-EXT^a^	NS^a^	SD
PAR	0.43 ^d^	0.6 ^d^	0.65 ^d^	0.51 ^d^	0.79 ^d^	0.78 ^d^	0.69 ^d^	0.54 ^d^
RH	−0.17 ^d^	−0.39 ^d^	−0.55 ^d^	−0.45 ^d^	−0.5 ^d^	−0.23 ^d^	−0.44 ^d^	−0.45 ^d^
TA	0.28 ^d^	0.45 ^d^	0.65 ^d^	0.36 ^d^	0.45 ^d^	0.31 ^d^	0.59 ^d^	0.31 ^d^
SWC	−0.24 ^d^	−0.11 ^d^	0.17 ^b^	0.14 ^d^	−0.09 ^b^	0.02 ns	−0.29 ^d^	na
u_*_	0.48 ^d^	0.5 ^d^	0.51 ^d^	0.45 ^d^	0.48 ^d^	0.27 ^d^	0.34 ^d^	0.09 ^d^
ET	0.42 ^d^	0.44 ^d^	0.62 ^d^	0.5 ^d^	0.79 ^d^	0.74 ^d^	0.7 ^d^	0.54 ^d^
GPP	0.46 ^d^	0.27 ^d^	0.48 ^d^	0.38 ^d^	0.55 ^d^	0.62 ^d^	0.6 ^d^	0.29 ^d^
TSEOP	−0.14 ^d^	0.1 ^d^	−0.03 ns	0.15 ^d^	−0.03 ns	0.04 ns	−0.05 ^d^	0.1 ^d^
*n*	396	1519	156	3767	418	447	15697	1179

	Deposition							
	BF	MO	HF	VA	OS-INT^a^	OS-EXT^a^	NS^a^	SD
PAR	−0.15ns	−0.29 ^d^	−0.09 ns	−0.11 ^d^	−0.54 ^d^	−0.02 ns	−0.17 ^d^	−0.02 ns
RH	0.33 ^d^	−0.11 ^d^	0.28 ^b^	−0.22 ^d^	0.18ns	−0.19 ns	0.27 ^d^	−0.07 ^b^
TA	−0.03 ns	−0.02 ns	−0.11 ns	−0.16 ^d^	−0.22 ^b^	0.14 ns	−0.32 ^d^	−0.17 ^d^
SWC	0.17 ns	−0.03 ns	−0.12 ns	−0.13 ^d^	0.09 ns	−0.03 ns	0.19 ^d^	na
u_*_	−0.3 ^d^	−0.46 ^d^	0.02 ns	−0.44 ^d^	−0.28 ^d^	−0.06 ns	−0.39 ^d^	−0.28 ^d^
ET	−0.12 ns	−0.29 ^d^	−0.1 ns	−0.16 ^d^	−0.46 ^d^	0.05 ns	−0.17 ^d^	−0.11 ^d^
GPP	−0.17 ns	−0.23 ^d^	−0.15 ns	−0.14 ^d^	−0.51 ^d^	−0.1 ns	−0.18 ^d^	−0.08 ^b^
TSEOP	−0.18 ns	0.1 ^d^	−0.01 ns	0.22 ^d^	−0.09 ns	−0.06 ns	−0.03 ns	0.03 ns
*n*	65	978	64	4917	72	45	1930	673

a excluding data influenced by site management

b *p* < 0.05

c *p* < 0.01

d *p* < 0.001

ns – not significant, na – not available).

**Table 4:** Variance explained (partial eta-squared, *η*^2^) in methanol emission and deposition based on univariate analysis of variance (UNIANOVA) using all data exclusive of Blodgett Forest and the grassland site data influenced by management activities. See Table 3 for abbreviations.

	*η*^2^ (%)	
	Emission	Deposition
Corrected model	56.84^d^	38.09^d^
Offset	0.09^d^	0.01 ns
PAR	0.69^d^	0.00 ns
TA	0.24^d^	0.02 ns
RH	0.06^d^	0.02 ns
u_*_	0.16^d^	0.03 ns
GPP	0.17^d^	0.00 ns
TSEOP	0.00 ns	0.00 ns
ET	0.11^d^	0.00 ns
Site	0.76^d^	2.96^d^
Site × PAR	0.58^d^	0.07 ns
Site × TA	0.79^d^	1.49^d^
Site × RH	1.45^d^	2.71^d^
Site × u_*_	0.29^d^	0.71^d^
Site × GPP	0.98^d^	0.01 ns
Site × TSEOP	0.38^d^	0.10 ns
Site × ET	0.22^d^	0.21^c^
*n*	23453	9092
