# Supplementary material for: An ecosystem-scale perspective of the net land methanol flux: synthesis of micrometeorological flux measurements
Source: Atmos Chem Phys. Author manuscript; Available in PMC 2015 May 14. (PMC4430827; doi:10.5194/acp-15-7413-2015)
Supplement: SM [file NIHMS62479-supplement-SM.docx]

Supplementary Material: An ecosystem-scale perspective of the net land methanol flux: synthesis of micrometeorological flux measurements

G. Wohlfahrt^1,2^, C. Amelynck^3^, C. Ammann^4^, A. Arneth^5^, I. Bamberger^5,6^, A. H. Goldstein^7^, L. Gu^8^, A. Guenther^9^, A. Hansel^10^, B. Heinesch^11^, T. Holst^12^, L. Hörtnagl^6^, T. Karl^13^, Q. Laffineur^14^, A. Neftel^4^, K. McKinney^15^, J. W. Munger^15^, S. G. Pallardy^16^, G. W. Schade^17^, R. Seco^18^, N. Schoon^3^

[1]{(Institute of Ecology, University of Innsbruck, Innsbruck, Austria)}

[2]{(European Academy of Bolzano, Bolzano, Italy)}

[3]{(Belgian Institute for Space Aeronomy, Brussels, Belgium)}

[4]{(Research Station Agroscope, Climate and Air Pollution Group, Zürich, Switzerland)}

[5]{(Karlsruhe Institute of Technology, IMK-IFU, Garmisch-Partenkirchen, Germany)}

[6]{(Institute of Agricultural Sciences, ETH Zürich, Zürich, Switzerland)}

[7]{(Department of Environmental Science, Policy, and Management, University of California, Berkeley, CA, USA)}

[8]{(Environmental Sciences Division, Oak Ridge National Laboratory, Oak Ridge, TN, USA)}

[9]{(Atmospheric Sciences and Global Change Division, Pacific Northwest National Laboratory, Richland, WA, USA)}

[10]{(Institute of Ion Physics and Applied Physics, University of Innsbruck, Innsbruck, Austria)}

[11]{(Exchanges Ecosystems-Atmosphere, Department Biosystem Engineering (BIOSE), University of Liege, Gembloux, Belgium)}

[12]{(Department of Physical Geography and Ecosystem Science, Lund University, Lund, Sweden)}

[13]{(Institute of Meteorology and Geophysics, University of Innsbruck, Innsbruck, Austria)}

[14]{(Royal Meteorological Institute, Brussels, Belgium)}

[15]{(School of Engineering and Applied Sciences, Harvard University, Cambridge, MA, USA)}

[16]{(Department of Forestry, University of Missouri, Columbia, MO, USA)}

[17]{(Department of Atmospheric Sciences, Texas A&M University, College Station, TX, USA)}

[18]{(Department of Earth System Science, University of California, Irvine CA 92697, USA)}

Correspondence to: G. Wohlfahrt (georg.wohlfahrt@uibk.ac.at)

Table S1. Details on setup and instrumentation for methanol flux measurements.

|  | **Blodgett Forest** | **Missouri Ozark** | **Harvard Forest** | **Vielsalm** | **Oensingen**  **INT** | **Oensingen**  **EXT** | **Neustift** | **Stordalen**  **mire** |
| --- | --- | --- | --- | --- | --- | --- | --- | --- |
| Flux method | REA | DEC | DEC | DEC | DEC | DEC | DEC | DEC |
| vDEC sampling interval and dwell time(s) | - | 1.2 (0.1) | 3.0 (0.2) | 2.0 (0.2) | 0.7-1.3 (0.2) | 0.7-1.3 (0.2) | 1.8-3.0 (0.2-0.5) | 3.0 (0.5) |
| Sonic anemometer model | CSAT, Campbell Scientific, USA | CSAT, Campbell Scientific, USA | SATI-K probe,  Applied Technologies, USA | R2,Gill UK | HS, Gill, UK | HS, Gill, UK | R3, Gill, UK | USA-1, Metek, Germany |
| PTR-MS | - | Ionicon Analytik, Austria | Ionicon Analytik, Austria | Ionicon Analytik, Austria | Ionicon Analytik, Austria | Ionicon Analytik, Austria | custom-built | Ionicon Analytik, Austria |
| Inlet length/inner diameter (m) | - | 40/0.0064 | 50/0.006 | 60/0.0064 | 30/0.0035 | 30/0.0035 | 14/0.004 | 12/0.008 |
| Inlet heating (Y/N) | - | N | N | Y | N | N | Y | Y |
| Inlet flow (l/min) | - | 10 | 11.8 | 9 | 4 | 4 | 8 | 20 |
| PTR-MS drift tube pressure (mbar) | - | 2.3 | 2.1 | 2.1 | 2.1 | 2.1 | 2.15-2.3 | 2.2 |
| PTR-MS drift tube voltage (V) | - | 540 | 600 | 600 | 550 | 550 | 550-600 | 600 |
| E/N (Td) | - | 110 | 126 | 143 | 122 | 122 | 130 | 130 |
| Key reference | Schade and Goldstein (2001) | Seco et al. (in preparation) | McKinney et al. (2011) | Laffineur et al. (2012) | Brunner et al. (2007) | Brunner et al. (2007) | Hörtnagl et al. (2011) | Holst et al. (2010) |


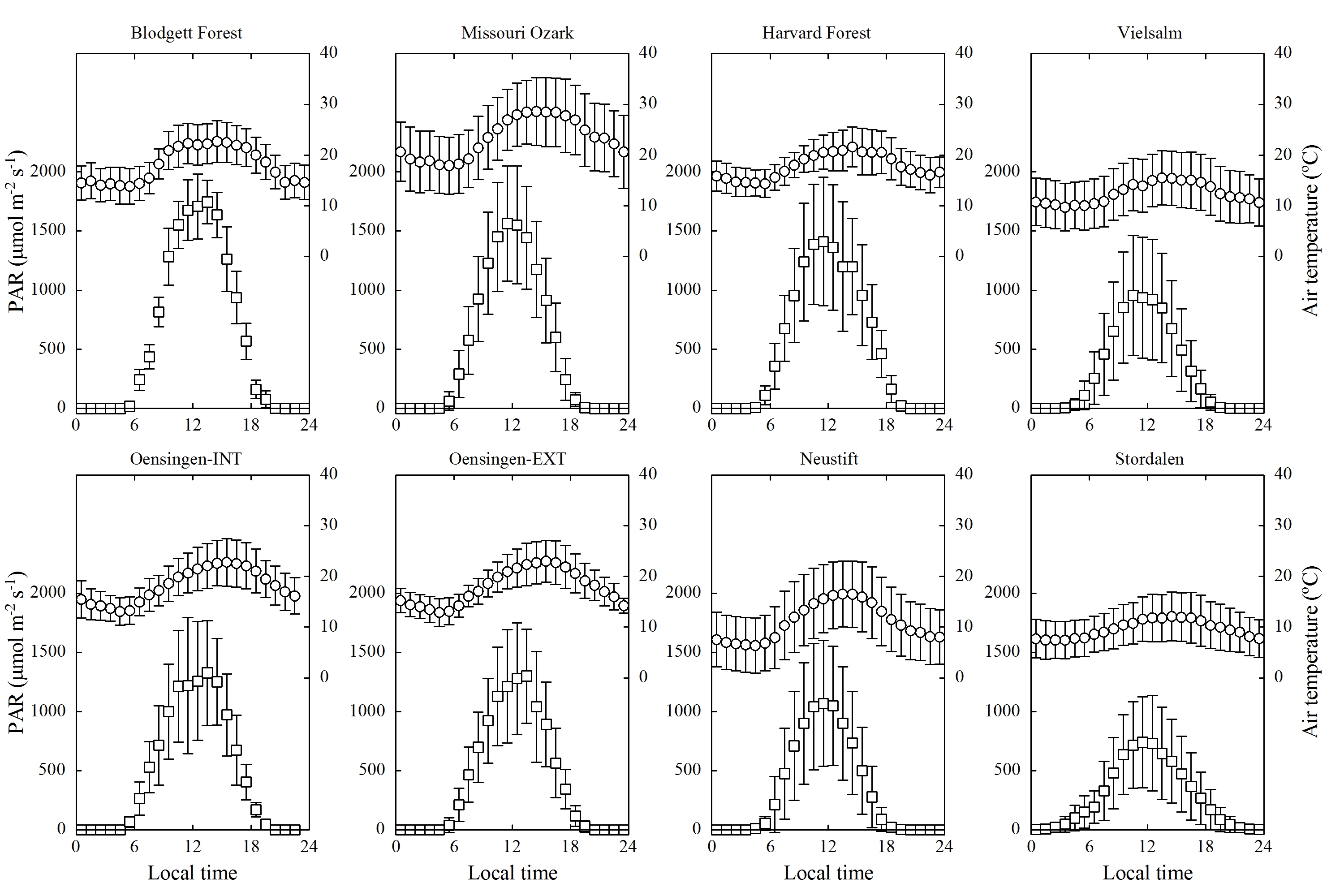


Figure S1. Hourly bin-averaged diurnal variation of photosynthetically active radiation (PAR; squares; left y-axis) and air temperature (circles; right y-axis) at the eight study sites (error bars represent ± one standard deviation).


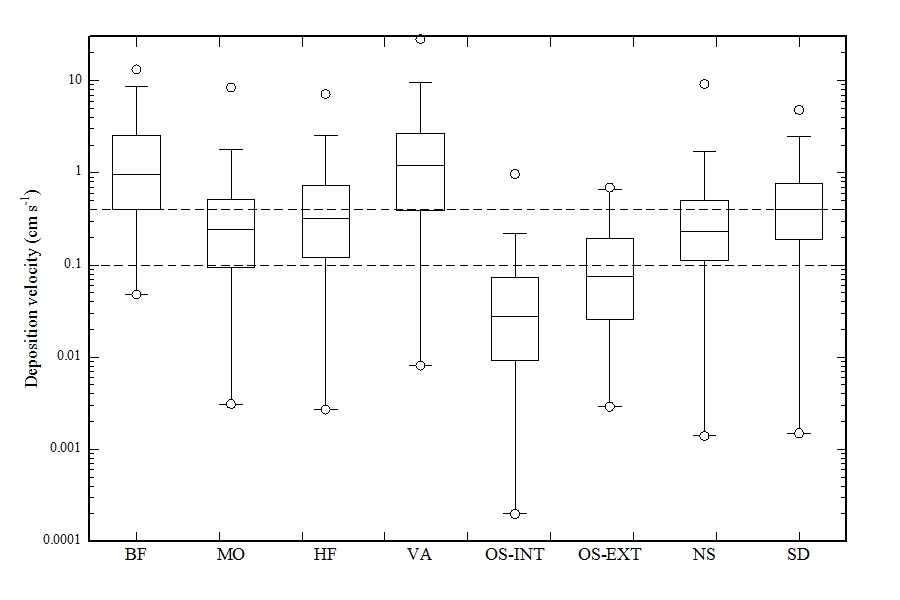


Figure S2. Box-plots of methanol deposition velocities at the eight study sites. Horizontal dashed lines indicate the range of deposition velocities (0.1-0.4 cm s^-1^) used in global budgets (see also Table 2). Box plots show minima/maxima (circles), 5% and 95% quartiles (whiskers), the interquartile range (box) and the median (horizontal line).


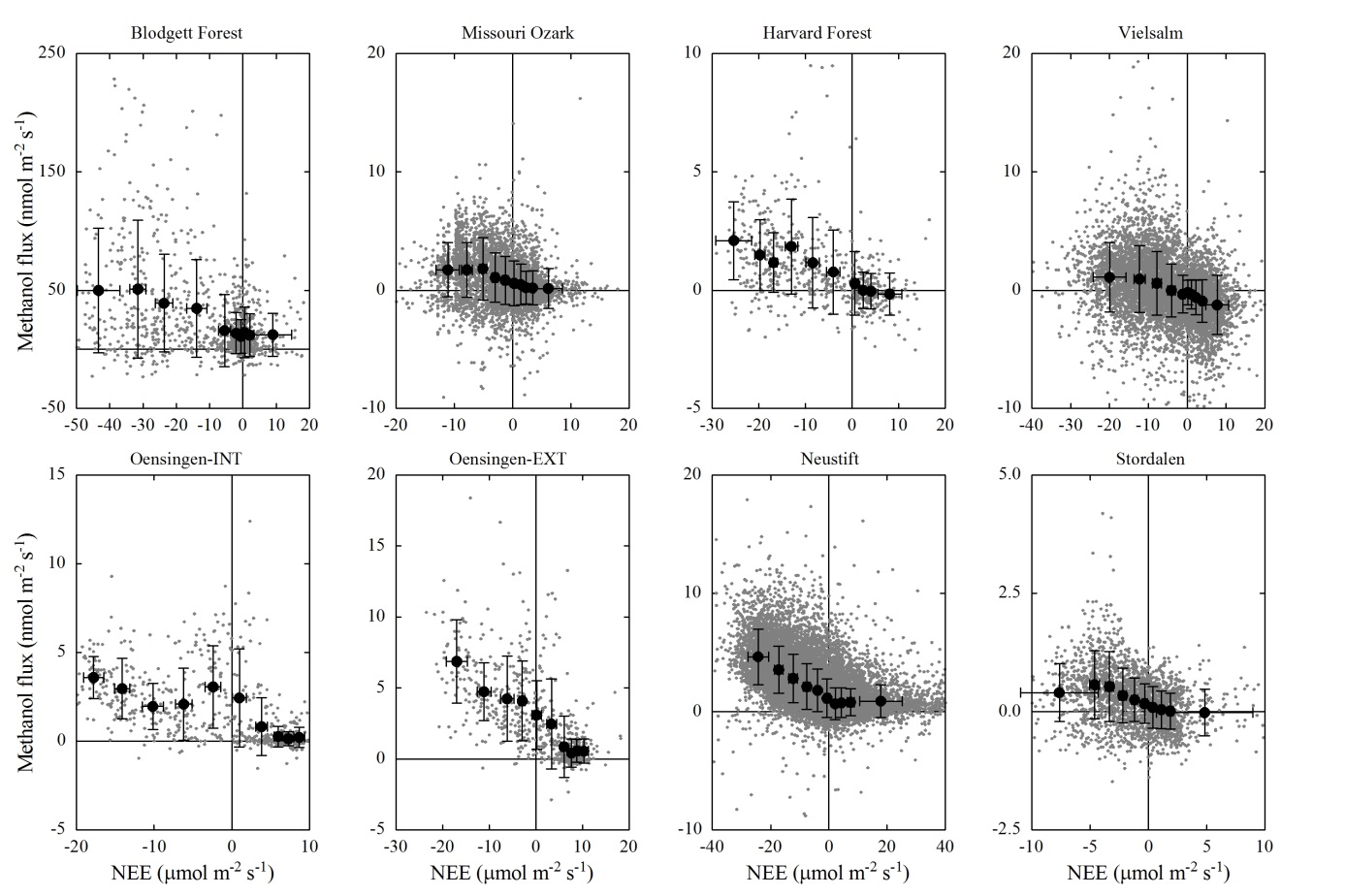


Figure S3. Relationship between the net ecosystem CO_2_ exchange (NEE) and methanol flux. Small grey symbols represent half-hourly flux measurements, black closed symbols 10 bin averages with equal numbers of data. Error bars refer to one standard deviation. Note different x- and y-scales in different panels.
